# *Clostridioides difficile* Infection in Central Asia: Current Evidence, Molecular Epidemiology, and Surveillance Priorities—A Narrative Review

**DOI:** 10.3390/pathogens15080853

**Published:** 2026-08-14

**Authors:** Dilshat Zamirovich Mukhamejanov, Alyona Vladimirovna Lavrinenko, Abdurakhim Toychiev, Fazliddin Khayriddin ogli Gaybullayev

**Affiliations:** 1 Scientific Research Laboratory, Karaganda Medical University, 12 Orskaya Street, Karaganda 100022, Kazakhstan; lavrinenko@qmu.kz; 2 Department of Infectious Diseases and Phthisiology, Karaganda Medical University, 12 Orskaya Street, Karaganda 100022, Kazakhstan; 3 Republican Specialized Scientific and Practical Medical Center of Epidemiology, Microbiology, Infectious and Parasitic Diseases, 2 Zakovat Street, Tashkent 100133, Uzbekistan; abdurahim1988@gmail.com; 4 Department of Infectious Diseases and Pediatric Infectious Diseases, Tashkent State Medical University, 2 Farabi Street, Tashkent 100109, Uzbekistan; fazliddin.xayriddin@mail.ru

**Keywords:** *Clostridioides difficile*, Central Asia, surveillance, antimicrobial stewardship, molecular epidemiology, ribotype 017

## Abstract

*Clostridioides difficile* infection (CDI) is a leading cause of healthcare-associated diarrhoea and a marker of antimicrobial exposure and healthcare quality, yet Central Asia is almost absent from the indexed literature. We reviewed available evidence on CDI epidemiology, antimicrobial use and molecular typing across Kazakhstan, Uzbekistan, Kyrgyzstan, Tajikistan and Turkmenistan, searching PubMed/MEDLINE, Google Scholar and accessible regional sources, and identified priorities for surveillance development. The retrievable evidence is sparse and fragmented. The strongest direct data came from Uzbekistan, where paediatric cohorts documented toxigenic *C. difficile* in up to 47% of the aetiological structure of antibiotic-associated diarrhoea in early-aged children. Nationwide surveys in Kazakhstan showed substantial antimicrobial use (38.2–40.2% of inpatients) and measurable healthcare-associated infection burdens (2.4–3.8%), indicating environments in which CDI may go unrecognised. No national CDI surveillance, ribotyping or resistance-gene data were identified for any of the five countries, whereas evidence from China, Thailand and other Asian settings indicates that ribotype 017 (sequence type 37) predominates in those countries. This absence appears more consistent with underdiagnosis, fragmented reporting and limited laboratory capacity than with true absence of disease. Sentinel surveillance, standardised diagnostic algorithms, integration with antimicrobial stewardship and infection prevention programmes, and regional typing collaborations are the practical priorities.

## 1. Introduction

*Clostridioides difficile* infection (CDI) is one of the leading causes of healthcare-associated diarrhoea worldwide and is associated with substantial morbidity, recurrence, mortality, prolonged hospitalisation and increased healthcare costs [1,2,3,4]. In many health systems, CDI is also treated as an indirect indicator of antimicrobial prescribing quality, infection prevention performance and diagnostic capacity [5,6,7].

In countries with established reporting systems, the CDI burden is monitored through standardised laboratory-confirmed frameworks. Even in well-resourced settings, however, cases are missed because clinical suspicion is inconsistent, testing practices are heterogeneous and laboratory access varies. The multinational EUCLID study demonstrated substantial under-ascertainment of CDI across European hospitals, and established the more general point that an absence of reported cases does not establish an absence of disease [8].

Over the past decade, awareness of CDI in Asia has grown, although the evidence remains unevenly distributed. Epidemiological and molecular data are considerably stronger in East Asia and parts of the Western Pacific, where ribotype 017 (multilocus sequence type 37) has repeatedly been identified as the dominant circulating lineage, distinct from the hypervirulent ribotype 027 strains that drive outbreaks in North America and Europe [9,10,11,12,13,14,15]. Whole-genome phylogeographic analyses indicate that ribotype 017 has circulated between Asia and Europe since at least the mid-twentieth century, with regional sublineages carrying distinct resistance determinants, particularly against fluoroquinolones, clindamycin and erythromycin [14,16]. Despite this growing regional picture, several geographic areas, including Central Asia, remain poorly characterised.

Central Asia—Kazakhstan, Uzbekistan, Kyrgyzstan, Tajikistan and Turkmenistan—is largely absent from the indexed CDI literature, despite the presence of recognised risk factors including antimicrobial exposure, healthcare-associated transmission, hospitalisation pressures and uneven diagnostic access. No ribotyping, sequence-typing or resistance-gene data have been published for any of the five countries. This is a consequential gap given the geographic proximity and the historical population and trade connectivity between Central Asia, the Russian Federation and East Asia, all regions in which CDI molecular epidemiology has been more thoroughly characterised.

The objective of this narrative review was to summarise the currently available evidence on CDI epidemiology, antimicrobial-use context and molecular characterisation relevant to Central Asia, and to identify practical priorities for surveillance development.

## 2. Materials and Methods

### 2.1. Study Design and Rationale

This work was conducted as a narrative review rather than a systematic review, and the reasons for that choice bear stating explicitly, since they are themselves informative about the state of the field.

Systematic methodology presupposes a body of comparable primary studies from which pooled estimates can be derived. Preliminary scoping identified no multicentre incidence studies, no standardised case definitions applied across settings, and no typing datasets from any of the five countries. Under these conditions, a systematic protocol would have returned a near-empty synthesis while excluding precisely the contextual evidence—antimicrobial-use surveys, healthcare-associated infection prevalence data and paediatric antibiotic-associated diarrhoea cohorts—that permits any inference about the region at all. A narrative approach was therefore selected as the design appropriate to the available evidence, with the aim of summarising and critically interpreting it rather than pooling it.

No review protocol was registered, and no PROSPERO entry was made, consistent with the narrative design.

### 2.2. Information Sources and Search Strategy

Literature searches were conducted up to April 2026. No lower date limit was applied, so that older regional reports would not be excluded a priori; in practice, the earliest source meeting the eligibility criteria was published in 2012. The primary databases searched were PubMed/MEDLINE and Google Scholar. Because indexed coverage of Central Asian clinical research is incomplete, database searching was supplemented by two further routes: hand-searching of the reference lists of all retrieved articles and reviews, and forward citation tracking of the regionally relevant papers identified in that way. National and regional journals published in Kazakhstan and Uzbekistan were searched directly where they were reachable by these routes; sources that could not be located in full text were not included.

Search terms were combined with Boolean operators and comprised: *Clostridioides difficile*, *Clostridium difficile*, CDI, pseudomembranous colitis, antibiotic-associated diarrhoea, ribotype, sequence type, molecular epidemiology, toxigenic culture, healthcare-associated infection and antimicrobial consumption, together with the geographic terms Central Asia, Kazakhstan, Uzbekistan, Kyrgyzstan, Tajikistan and Turkmenistan. Equivalent Russian-language terms were used when searching regional sources.

No language restriction was applied at the search stage. Records in English and Russian were assessed in full; Russian-language regional publications were retained where they contained extractable data, and one such source is included among the studies reviewed. Records in languages that the review team could not assess were noted but not included, and this is acknowledged as a limitation.

### 2.3. Eligibility Criteria

Sources were eligible if they contained extractable information relating to at least one of the following:

- Direct CDI epidemiology, including incidence, prevalence, recurrence or mortality;
- Antibiotic-associated diarrhoea in which *C. difficile* was investigated as an aetiological agent;
- Antimicrobial use relevant to CDI risk;
- Healthcare-associated infection burden relevant to the interpretation of CDI;
- Molecular typing, ribotype or sequence-type data from Central Asia or from epidemiologically connected regions;
- Diagnostic or surveillance capacity relevant to CDI detection.

Sources were excluded if they were purely narrative commentaries without extractable data, if they duplicated a dataset already included, or if the relationship to *C. difficile* could not be established from the report. Case reports were not excluded a priori, given the sparsity of the evidence base, but none contributing extractable regional data was identified.

Screening of titles, abstracts and full texts was carried out collaboratively by two authors (D.Z.M. and A.V.L.) working jointly rather than independently in duplicate, and any uncertainty over eligibility was resolved by discussion among all authors. The absence of independent duplicate screening is acknowledged in Section 4.4. Because quantitative synthesis was not undertaken, formal risk-of-bias assessment and certainty-of-evidence grading were not applied; instead, the design and limitations of each included source are described narratively in the Results.

## 3. Results

### 3.1. Overall Evidence Base

The most notable finding was the marked scarcity of direct CDI epidemiological studies from Central Asia. No multicentre adult incidence studies, national surveillance reports, recurrence datasets, ribotyping or sequence-typing data, or standardised regional burden estimates were identified.

This contrasts with Europe and North America, where structured reporting systems and multicentre networks have substantially improved burden estimation [5,6,8], and with East Asia, where molecular typing networks have characterised the dominant ribotype 017 lineage across multiple countries [9,12,14,15].

### 3.2. Kazakhstan

No CDI-specific epidemiological studies from Kazakhstan were identified in the reviewed literature. However, several recent national investigations provide clinically relevant contextual evidence.

A multicentre point-prevalence survey in four acute-care hospitals involving 701 patients reported healthcare-associated infections in 3.8% of hospitalised patients. Nearly half of the identified infections occurred in intensive care settings, where CDI risk may be elevated because of broad-spectrum antibiotic exposure, severity of illness, prolonged hospitalisation and contact transmission [17].

A subsequent nationwide survey across 26 hospitals including 8076 patients reported an overall healthcare-associated infection prevalence of 2.4%. Antimicrobial use was documented in 40.2% of inpatients, with cephalosporins the most frequently prescribed class. Broad-spectrum cephalosporin exposure is a recognised risk factor for CDI [18].

A separate national evaluation of antibiotic consumption between 2019 and 2023 demonstrated substantial population-level antimicrobial use, peaking during the pandemic period [19].

Taken together, these findings do not quantify the CDI burden, but they describe healthcare and prescribing conditions compatible with CDI occurrence that may not be captured. Kazakhstan may also possess the strongest operational basis in the region for future sentinel CDI monitoring, because multicentre prevalence studies have already been implemented successfully.

### 3.3. Uzbekistan

The clearest directly relevant evidence in the region originated from Uzbekistan.

A paediatric hospital-based study from Tashkent involving children aged 1 month to 3 years reported antibiotic-associated diarrhoea in 25.0% of children receiving antibiotics, with toxigenic *C. difficile* identified in 20.8% of affected cases [20].

A further paediatric study published in 2024 reported that *C. difficile* accounted for 47% of the aetiological structure of antibiotic-associated diarrhoea among early-aged children [21].

Although these studies were confined to paediatric cohorts and cannot be extrapolated to the national adult burden, they provide direct microbiological evidence that CDI occurs in routine clinical practice within Central Asia. Uzbekistan therefore offers the strongest published signal of recognised CDI activity in the region. Neither study included strain typing, so the ribotypes circulating in Uzbekistan remain entirely uncharacterised.

### 3.4. Kyrgyzstan, Tajikistan and Turkmenistan

No indexed primary studies with extractable CDI epidemiological or molecular data were identified from Kyrgyzstan, Tajikistan, or Turkmenistan.

This should not be read as confirmed absence of disease. It more plausibly reflects constraints on diagnostic access, limited reporting infrastructure, publication invisibility or under-ascertainment.

### 3.5. Molecular Epidemiology Context from Neighbouring Regions

Although no typing data exist for Central Asia, context from epidemiologically connected areas is informative. *C. difficile* ribotype 017 (multilocus sequence type 37) is established as the dominant epidemic lineage across East Asia, including China, South Korea and Thailand, in contrast to the ribotype 027 strains that predominate in North America and parts of Europe [9,12,14,15].

Hospital-based typing studies from China have consistently identified ribotype 017/ST37 among the predominant genotypes, both in early surveys that first characterised the lineage as an under-ascertained regional problem and in subsequent larger series from eastern and southwestern centres [22,23,24,25]. Comparable, if more limited, evidence has been reported from Southeast Asia, where single-centre and multicentre studies have documented ribotype 017 circulation alongside considerable genotypic diversity [26,27]. Two features of this body of work are relevant here. First, in nearly every setting the identification of a dominant lineage followed rather than preceded the establishment of routine diagnostic testing, indicating that strain characterisation becomes achievable once case ascertainment is established. Second, several of these studies were conducted in middle-income health systems [25,26,27], which suggests that a first typing study in Central Asia need not await the construction of a national network.

High-resolution phylogenomic analyses estimate that ribotype 017 began disseminating globally between 1953 and 1983, with acquisition of an erythromycin–clindamycin resistance transposon identified as a key factor in its emergence following the introduction of clindamycin into clinical practice [14]. Ribotype 017 isolates frequently carry resistance determinants against fluoroquinolones and macrolide–lincosamide–streptogramin agents, both of which are widely used across Central Asian health systems [16,18,19].

Given the documented historical and contemporary population, trade and healthcare connectivity between Central Asia, the Russian Federation and East Asia, it is biologically plausible that ribotype 017 or related multidrug-resistant sublineages circulate within Central Asian healthcare facilities. In the complete absence of regional isolate collections, however, this remains a hypothesis derived from geographic proximity and regional epidemiological patterns. It requires direct investigation and should not be treated as an established finding. The available country-level evidence is summarised in Table 1.

Read across its rows, Table 2 makes the structure of the regional gap explicit. For China and Thailand, all three descriptive columns can be populated from published isolate collections; for North America and Europe a different lineage is documented with equal confidence. For Central Asia every column is empty, and the reason differs in kind from the others: these are not fields in which conflicting or low-quality data exist, but fields for which no measurement has ever been attempted. The contrast between the third and fourth rows is the analytically important one. Because the dominant lineage differs between Asia and the West, the identity of the lineage is demonstrably a property that varies geographically, and therefore it is not a property that can be transferred to Central Asia by inference from its neighbours. What would populate the fourth row is modest in scale: a consecutive series of toxigenic isolates from even a single tertiary centre, characterised by ribotyping or whole-genome sequencing, would convert the region from a blank in the regional picture into a data point that can be compared with the rows above it.

## 4. Discussion

This review shows that Central Asia remains a major blind spot in the global epidemiology of *Clostridioides difficile* infection. The available evidence is more consistent with under-ascertainment, fragmented reporting systems, limited diagnostic capacity and publication gaps than with a confirmed absence of disease.

This interpretation is not unique to the region. Even in Europe, where reporting systems are comparatively mature, substantial underdiagnosis emerged once standardised case-finding was applied [8]. Low published incidence in poorly studied settings should therefore be interpreted with corresponding caution.

Kazakhstan appears particularly important from a regional public health perspective. Although no CDI-specific studies were identified, recent nationwide healthcare-associated infection and antimicrobial-use surveys demonstrate measurable inpatient transmission burdens and substantial antibiotic exposure [17,18,19]. These conditions are biologically compatible with ongoing but insufficiently measured CDI. Because Kazakhstan has already demonstrated the capacity to conduct multicentre hospital prevalence studies, it may represent the most practical starting point for regional initiatives.

Uzbekistan provides the strongest direct published evidence of CDI in Central Asia. Paediatric cohorts documenting toxigenic *C. difficile* in up to 47% of the aetiological structure of antibiotic-associated diarrhoea indicate that laboratory-confirmed disease is already present in routine clinical settings [20,21]. These findings should prompt broader hospital-based investigation, including in adult populations, rather than being treated as isolated paediatric observations.

The complete absence of typing data represents a further and previously unaddressed gap. Regional evidence from China and Thailand shows that ribotype 017 is the dominant circulating lineage across much of Asia, frequently carrying resistance to fluoroquinolones and macrolide–lincosamide–streptogramin agents [9,12,14,15]—precisely the classes documented as heavily used across Central Asian health systems [18,19]. Without typing data, it is not possible to determine whether CDI in Central Asia, where it occurs, reflects regionally connected ribotype 017 transmission, locally distinct strains, or a heterogeneous mixture. Establishing even a small pilot isolate collection with ribotyping or whole-genome sequencing at a single reference centre would be a high-yield first step.

From a practical standpoint, phased sentinel monitoring in tertiary hospitals may represent the most realistic next step. Such programmes could initially focus on selected referral hospitals with microbiology capacity, standardised case definitions and structured reporting. International guidance supports multistep diagnostic approaches combining sensitive screening assays, toxin confirmation and clinical correlation [7,28,29,30].

### 4.1. Practical Barriers to CDI Diagnosis and Surveillance in Central Asian Healthcare Systems

Any realistic appraisal of CDI monitoring in Central Asia must begin with the diagnostic infrastructure that would have to support it. The barriers involved are not unique to this region, but they are consequential enough that a framework designed without reference to them is unlikely to survive contact with routine practice.

The first constraint is laboratory capacity. Guideline-concordant CDI diagnosis relies on a multistep algorithm combining a sensitive screening assay, such as glutamate dehydrogenase detection or nucleic acid amplification testing, with toxin confirmation and clinical correlation [28,29]. Implementing such an algorithm requires validated commercial assays, reagent supply chains that tolerate irregular procurement cycles, and technologists trained in their interpretation. Toxigenic culture and isolate recovery—indispensable if any typing is ever to follow—additionally require anaerobic incubation capacity, selective media and cycloserine–cefoxitin–fructose agar, none of which forms part of the routine repertoire of most district-level laboratories in the region. Where such capacity exists, it is typically concentrated in a small number of national or tertiary institutions.

The second constraint is cost, and it operates at two levels. Per-test costs for toxin immunoassays and nucleic acid amplification remain substantially higher than for the stool cultures that dominate routine enteric diagnostics; and in health systems where laboratory budgets are allocated prospectively rather than reimbursed per episode, adding an assay for a condition with no recorded local burden is difficult to justify. This produces a self-reinforcing loop that deserves explicit recognition: testing is not funded because no burden has been demonstrated, and no burden can be demonstrated because testing is not funded. Breaking that loop generally requires an external stimulus—a time-limited pilot, a research grant or an externally supported sentinel programme—rather than incremental budgetary adjustment.

The third constraint is pre-analytical. *C. difficile* toxins degrade at ambient temperature, and delays between stool passage and laboratory processing reduce assay sensitivity. Where specimens are transported between facilities without a maintained cold chain, false-negative results may be common and, critically, invisible: a negative result generated from a degraded specimen is indistinguishable in the laboratory record from a true negative. Any sentinel protocol should therefore specify a maximum permissible transport interval and require documentation of internal quality control for the assays used, so that negative results can be interpreted with appropriate confidence.

The fourth constraint is the workforce. Case ascertainment for CDI depends less on laboratory technology than on clinical suspicion, and clinical suspicion depends on the presence of infection prevention and control practitioners and clinical microbiologists able to prompt testing in patients with healthcare-onset diarrhoea. Where these roles are thinly staffed, or where diarrhoea in hospitalised patients is managed empirically as an expected consequence of antibiotic therapy, testing will not be requested regardless of whether the assay is available.

Finally, CDI competes for attention with tuberculosis, viral hepatitis, HIV and antimicrobial resistance, all of which carry established national programmes, dedicated funding and international reporting obligations in the region [31,32]. A proposal that positions CDI monitoring as an additional vertical programme is unlikely to be adopted. One that positions it as an incremental extension of infection prevention and antimicrobial resistance activity that already exists has a considerably better prospect.

### 4.2. Implementation Considerations for the Proposed Framework

**Figure 1:**
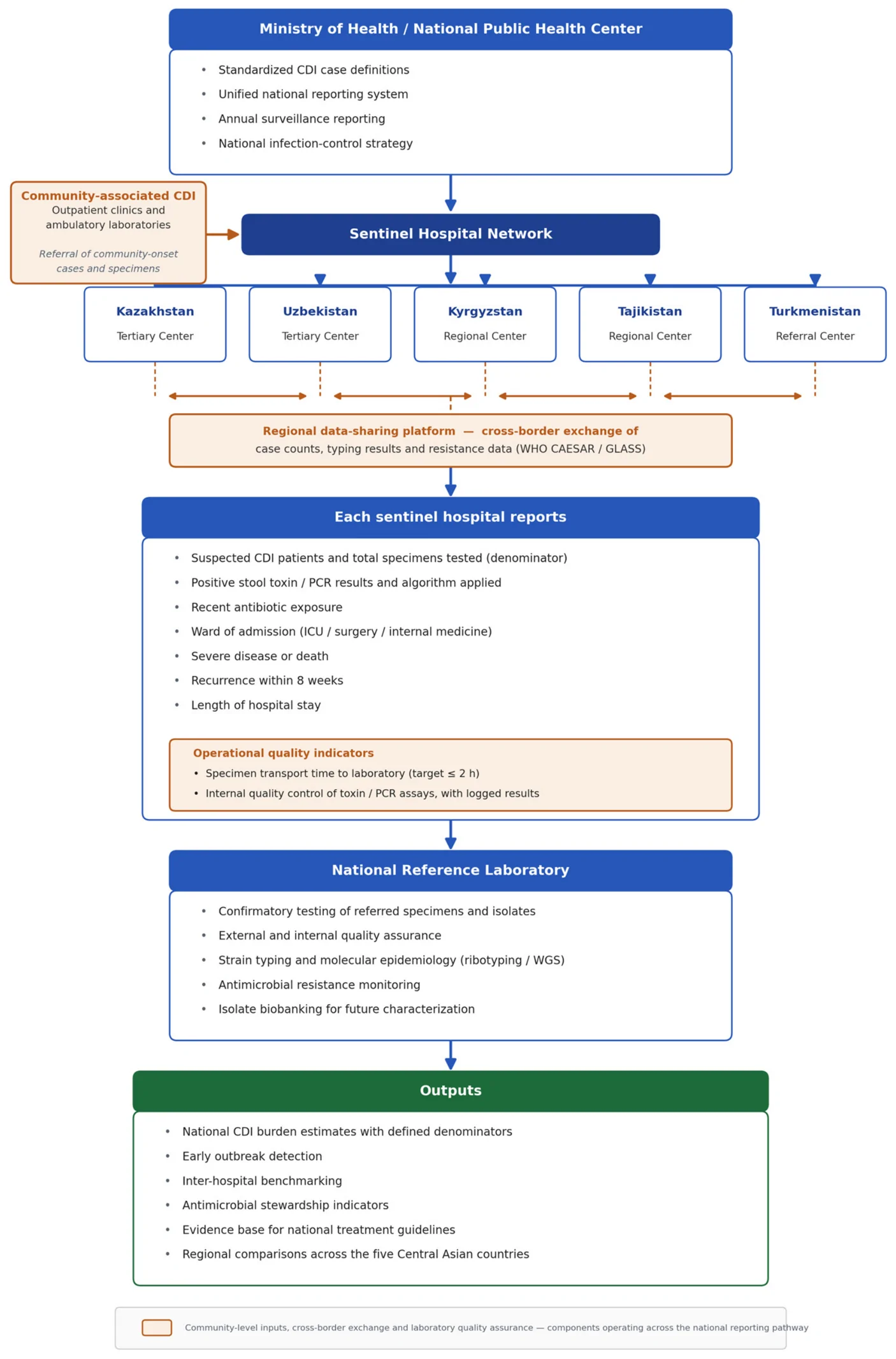
Proposed national and regional CDI surveillance framework for Central Asia.

A practical national framework, incorporating ministry-level governance, a sentinel hospital network, standardised case reporting and reference-laboratory support, is presented in Figure 1. The framework is deliberately schematic, and it is reasonable to ask how such a structure could be established in practice. Several existing mechanisms make a phased approach more tractable than building from scratch.

The most directly relevant is the Central Asian and European Surveillance of Antimicrobial Resistance network (CAESAR), a joint initiative of the WHO Regional Office for Europe, the European Society of Clinical Microbiology and Infectious Diseases, and the Dutch National Institute for Public Health and the Environment, which operates as a regional component of the WHO Global Antimicrobial Resistance and Use Surveillance System (GLASS) [32,33]. All five Central Asian countries are enrolled in CAESAR, and several already submit national data to it [33]. CAESAR therefore provides much of the architecture that a CDI programme would otherwise have to create independently: an established reporting relationship between national laboratories and a regional coordinating centre, an external quality assessment scheme, agreed data governance arrangements and—importantly for the cross-border dimension—a forum in which participating countries already exchange outputs. Adding a defined CDI module to an existing CAESAR reporting stream is a materially smaller undertaking than establishing a parallel network.

A phased sequence follows from this. A first phase might involve two to four sentinel tertiary hospitals with existing microbiology capacity—plausibly in Kazakhstan and Uzbekistan, where multicentre point-prevalence work and direct microbiological detection of toxigenic *C. difficile* have respectively already been demonstrated [17,18,20,21]—applying a standardised two-step diagnostic algorithm and a common case-report form, with the explicit initial objective of establishing testing denominators rather than incidence estimates. A second phase would add isolate retention and shipment to a single designated reference laboratory for ribotyping or whole-genome sequencing, an approach for which single-centre typing studies in comparable settings provide a feasible precedent [27]. A third phase would extend participation to the remaining countries and formalise regional data exchange.

Reference laboratory capacity need not be built domestically at the outset. Twinning arrangements with established *C. difficile* reference centres, and participation in external quality assessment for typing, allow isolate characterisation to begin while local capacity develops, and have the additional advantage of embedding comparability with international datasets from the beginning. Regarding financing, the realistic sources are not CDI-specific: national antimicrobial resistance action plan budgets, WHO country cooperation programmes, multilateral development bank health-system strengthening loans and investigator-initiated research funding are all more plausible entry points than a dedicated CDI appropriation.

Data generated by such a programme would have clinical as well as epidemiological value. Where typing is not yet available, routinely collected inflammatory and haemogram-derived indices have been shown to assist with mortality risk stratification in patients with CDI [34], and interpretable machine learning models built on routine hospital records have been used to estimate individual CDI risk in hospitalised patients, with inflammatory markers such as C-reactive protein, interleukin-6 and neutrophil counts consistently ranking among the strongest predictors [35]. Both approaches are presented by their authors as supportive risk-stratification aids rather than diagnostic tools, and both draw on exactly the categories of structured clinical data—laboratory values, comorbidities, antibiotic exposure and length of stay—that a sentinel programme would generate as a by-product of case reporting. The latter study also notes that external validation across different healthcare systems and patient populations remains outstanding [35]. That caveat has direct regional relevance: settings without structured case ascertainment can neither contribute to such validation nor benefit from the resulting tools, so precision-medicine approaches to CDI are likely to reach Central Asia only once the reporting layer described here exists.

### 4.3. Wider Benefits

CDI monitoring should be integrated into antimicrobial stewardship and infection prevention programmes. Because CDI incidence reflects both antibiotic pressure and infection-control performance, it can serve as a system-level quality indicator [31,32]. Integration with GLASS reporting infrastructure, in which Uzbekistan and Kazakhstan already participate for other pathogens, would minimise the need for entirely new reporting architecture [32].

More broadly, strengthening CDI monitoring in Central Asia may deliver benefits beyond a single pathogen. Improved laboratory diagnostics, antimicrobial-use monitoring, typing capacity and hospital epidemiology systems would also support preparedness for other healthcare-associated infections and antimicrobial resistance threats.

The Ministry of Health or National Public Health Center establishes standardised case definitions, a unified reporting system, annual reporting and a national infection-control strategy. These policies are implemented through a sentinel hospital network comprising tertiary centres in Kazakhstan and Uzbekistan, regional centres in Kyrgyzstan and Tajikistan, and a referral centre in Turkmenistan. Two inputs feed the network: hospital-onset cases, and community-onset cases referred from outpatient clinics and ambulatory laboratories, so that community-associated CDI is not excluded by design. Each sentinel hospital reports suspected cases together with the total number of specimens tested, providing a denominator; positive stool toxin or PCR results and the diagnostic algorithm applied; recent antibiotic exposure; ward of admission; severe disease or death; recurrence within eight weeks; and length of stay. Two operational quality indicators are recorded alongside these variables—specimen transport time to the laboratory, with a target of two hours or less, and internal quality control of toxin and PCR assays—so that negative results can be interpreted with known analytical confidence. Participating countries exchange case counts, typing results and resistance data through a regional data-sharing platform aligned with the WHO CAESAR and GLASS reporting streams. Cases and isolates are forwarded to a National Reference Laboratory for confirmatory testing, quality assurance, strain typing, resistance monitoring and isolate biobanking. Outputs include national burden estimates with defined denominators, early outbreak detection, inter-hospital benchmarking, stewardship indicators, an evidence base for treatment guidelines, and regional comparisons across the five countries. Components shown in orange denote community-level inputs, cross-border data exchange and laboratory quality assurance; these operate across the national reporting pathway shown in blue rather than within a single tier of it.

### 4.4. Limitations

Several limitations should be considered when interpreting this review.

First, and most importantly for a region-focused synthesis, the accessibility of regional literature is uneven in ways that are difficult to quantify. A substantial proportion of clinical and epidemiological research in Central Asia is published in national journals that are not indexed in PubMed/MEDLINE, Scopus or Web of Science, and a further body of relevant work exists as dissertation abstracts, ministry of health reports, sanitary-epidemiological service bulletins and conference proceedings that are not systematically catalogued or, in many cases, not available online at all. Access to this grey literature is also unequal across the five countries: institutional repositories and open national reporting are more developed in Kazakhstan and Uzbekistan than in Kyrgyzstan, Tajikistan and Turkmenistan. The apparent gradient in available evidence across the region may therefore partly reflect differential visibility of national output rather than differential research activity, and the absence of identified studies from three of the five countries should be read with this caveat firmly in mind.

Second, and related, the language of publication introduces additional asymmetry. Relevant work published in Russian, Kazakh or Uzbek may be under-represented in searches conducted primarily through international databases, even where hand-searching of regional sources was undertaken.

Third, the included studies were heterogeneous in design, population, case definition and diagnostic method, which limits direct comparison between them and precludes pooled estimation. Quantitative synthesis and formal risk-of-bias assessment were therefore not undertaken.

Fourth, the molecular epidemiology presented here derives entirely from neighbouring regions rather than from Central Asian isolates. This material provides regional context only; it is hypothesis-generating and does not constitute evidence about strains circulating in Central Asia.

Fifth, as a narrative review, this work did not follow a registered protocol, and study selection was not performed in duplicate. Selection and interpretation therefore carry a degree of subjectivity that a systematic approach would have reduced.

Finally, publication bias and under-reporting are likely to operate in the same direction, both tending to understate the extent of recognised CDI activity in the region.

Notwithstanding these constraints, the scarcity of retrievable data is itself a substantive finding, and it defines the case for structured epidemiological and molecular investigation.

## 5. Conclusions

Published evidence on the epidemiology of CDI in Central Asia remains extremely limited.

Current data confirm that *Clostridioides difficile* disease occurs within the region, but no reporting framework defines its true burden, and no molecular data exist to characterise circulating strains. The absence of epidemiological and molecular estimates is more consistent with diagnostic and reporting gaps than with proven low incidence.

Establishing sentinel surveillance systems, improving laboratory capacity, standardising diagnostic algorithms, initiating regional typing collaborations and integrating CDI monitoring into antimicrobial stewardship and infection prevention programmes should be regarded as practical regional priorities. Several of these steps can be built onto reporting infrastructure that already exists rather than being created from nothing.

Without deliberate effort to generate reliable epidemiological and molecular data, CDI will remain under-ascertained and inadequately quantified across Central Asia.

## Figures and Tables

**Table 1:** Available evidence of *Clostridioides difficile* infection in Central Asia, by country.

Country	Direct CDI Studies	Key Evidence	Main Findings	Major Gaps	Interpretation
Kazakhstan	None CDI-specific	2024 multicentre PPS; 2025 national PPS; antibiotic use analysis	HAI prevalence 3.8% and 2.4%; AMU 40.2%; cephalosporins common	No incidence, recurrence, mortality or typing data	Compatible with CDI that is occurring and not captured
Uzbekistan	Yes (paediatric)	2018 paediatric study; 2024 paediatric AAD study	Toxigenic *C. difficile* in 20.8–47% of paediatric AAD cases	No adult data, no national reporting, no typing	Strongest direct regional evidence
Kyrgyzstan	None identified	No extractable indexed evidence	No verified national data	Diagnostics and reporting infrastructure absent	Likely evidence gap
Tajikistan	None identified	No extractable indexed evidence	No verified national data	Diagnostics and reporting infrastructure absent	Likely evidence gap
Turkmenistan	None identified	No extractable indexed evidence	No verified national data	Diagnostics and reporting infrastructure absent	Likely evidence gap

**Table 2:** Regional molecular epidemiology context: *C. difficile* ribotype 017 in Asia and implications for Central Asia.

Region	Dominant Lineage	Resistance Profile	Relevance to Central Asia
China (eastern, southwestern)	RT 017/ST 37	Fluoroquinolone, clindamycin and erythromycin resistance common	Geographic and trade connectivity; no comparable data exist for Central Asia
Thailand	RT 017 (MDR and non-MDR sublineages)	Multidrug-resistant clusters identified in tertiary hospitals	Demonstrates feasibility of single-centre typing studies in middle-income settings
North America/Europe	RT 027 (hypervirulent)	Fluoroquinolone resistance; binary toxin positive	Distinct epidemiology; shows that the dominant lineage varies by region and cannot be assumed
Central Asia (this review)	Unknown—no isolates typed	Not characterised	A complete molecular blind spot; no inference about circulating lineages is currently possible

## Data Availability

No new data were created or analyzed in this study. Data sharing is not applicable to this article.

## Abbreviations

AAD, antibiotic-associated diarrhoea; AMR, antimicrobial resistance; AMU, antimicrobial use; CAESAR, Central Asian and European Surveillance of Antimicrobial Resistance; CDI, *Clostridioides difficile* infection; ECDC, European Centre for Disease Prevention and Control; GLASS, Global Antimicrobial Resistance and Use Surveillance System; HAI, healthcare-associated infection; MLST, multilocus sequence typing; PPS, point-prevalence survey; RT, ribotype; ST, sequence type; WGS, whole-genome sequencing.
